# Transfer of sediment-derived carbon into aquatic plants for ^14^C biosphere assessment

**DOI:** 10.1007/s00411-026-01199-7

**Published:** 2026-02-05

**Authors:** Soroush Majlesi, Safi Ullah, Zahra Shirani, Jukka Pumpanen, Susanna Salminen-Paatero, Jarkko Akkanen, Ari T. K. Ikonen

**Affiliations:** 1 Department of Chemistry, Radiochemistry, University of Helsinki, P.O. Box 55 (A. I. Virtasen aukio 1), Helsinki, 00014 Finland; 2 Department of Environmental and Biological Sciences, University of Eastern Finland, P.O. Box 1627, Kuopio, FI-70211 Finland; 3 Department of Environmental and Biological Sciences, University of Eastern Finland, PO Box 111, Joensuu, FI-80101 Finland; 4 EnviroCase, Ltd, Käppärätie 9 A 18, Pori, 28120 Finland

**Keywords:** Radiocarbon, Radioecology, Hydrophytes, Organic matter, Aquatic ecosystem, Radioactive waste

## Abstract

Among the released radionuclides from nuclear power plants and radioactive waste repositories, ^14^C is of great importance. Continuous discharges of ^14^C from nuclear industries, the risk of uncontrolled releases, and possible leaking from facilities may cause a threat to the biosphere. Because of high mobility and a long half-life of ^14^C, it has great potential to be released into aquatic ecosystems and to be assimilated by aquatic plants. However, the amount of ^14^C incorporated into organic matter and hydrophytes is largely unknown. In this study, the uptake of carbon from sediment into aquatic plants was investigated in a microcosm experiment. The study was carried out based on the natural difference in the isotopic signature of ^14^C between the 8000-year-old peat and more enriched sources (water and atmosphere). The two-pool isotope mixing model was applied to determine the relative contribution of each source (sediment vs. air/water) to the hydrophytes. The results indicated the highest contribution of sediment-derived carbon to the free-floating *Lemna minor* (up to 60%), followed by submerged *Littorella uniflora* (15–17%) and the emergent species, *Stachys palustris* and *Lysimachia nummularia* (up to 10%). Despite the contribution of sediment-derived C to their C source, the hydrophytes incorporated less than 2% of their total C from sediment. The results also indicated the importance of floating plants in more efficient uptake of sediment-derived C available in water column or the air. Furthermore, no significant difference was found in the transfer of sediment-derived C between the roots and the leaves within the species.

## Introduction

Nuclear power is recognized as a globally important energy source and an appropriate replacement for the use of fossil fuels to mitigate the climate change crisis. In Finland, there are currently five nuclear power reactors in Olkiluoto and Loviisa, providing about 34% of the electricity demand (World Nuclear Association 2023). The fifth unit started its operation in 2023. For the isolation and management of radioactive wastes, Finland has selected geological disposal as the most efficient approach (Posiva 2012). However, radionuclides, discharged from nuclear power plants and waste repositories into the biosphere remain the major concern of the nuclear power industry. Although the present biosphere models on possible radionuclide transportation from geological repositories have predicted sufficient protection of humans and non-human biota (Kautsky et al. 2016), risks of unpredicted releases (e.g., accidents, failure in waste storage systems), and continuous discharges from reactors should not be neglected. Therefore, constant monitoring and radiological risk assessments are also warranted for radioactive wastes.

Among the radionuclides released from nuclear power plants and waste repositories, radiocarbon (^14^C) is of great importance. It is present in a large inventory of nuclear reactor production systems and waste materials (Amiro et al. 1991; Sheppard et al. 1991; Yim and Caron 2006; Limer et al. 2017). Importantly, ^14^C has biogeochemical characteristics identical to stable isotopes of carbon (C), (^12^C and ^13^C), and it easily incorporates into the C cycle. Carbon, in general, is a fundamental organic element essential for biota. Therefore, after the incorporation of ^14^C into food webs, it can effectively engage in the metabolisms of living organisms. Furthermore, its high mobility in the environment and long half-life (5370 years) increase its radioecological concern (Pérez-Sánchez et al. 2009).

In radioactive waste materials, the release of ^14^C occurs through gaseous (e.g., ^14^CO_2_ and ^14^CH_4_) and liquid-borne discharges or after the disposal of solid wastes (IAEA 2004; Yim and Caron 2006). Moreover, in geological repositories, ^14^C is mainly in the form of low-weight organic molecules such as acetate, higher molecular weight compounds (e.g., fulvic and humic acids), and inorganic C (CO_2_ or bicarbonate species). The produced ^14^CO_2_ then becomes available for plants for assimilation through photosynthesis (Avila and Pröhl 2008; Hoch 2014; Doulgeris et al. 2015). The fixed atmospheric C in plants is either taken up by grazing/herbivorous animals and humans, or it may leach into deeper soil layers, including soil solution and groundwater as dissolved or particulate organic or inorganic matter. This process occurs through root exudation as plants allocate fixed C towards roots for plant growth and root maintenance (Lange et al. 2015).

After plants’ lifespan, ^14^C from their leftovers (e.g., root or leaf litter) can be mixed with organic matter (Dias et al. 2009). By incorporating ^14^C into organic matter, some proportion can be taken up by the roots from underground sources, or it may escape back into the atmosphere via soil respiration after decomposition of organic matter by soil microbes (Mobbs et al. 2014; SKB 2019). This may result in re-assimilation of ^14^C by plants. Another pathway is the indirect uptake of ^14^CH_4_ in soil from underground facilities after microbial transformation of CO_2_ or low-weight organic molecules to CH_4_ and its subsequent oxidation to inorganic C in the presence of oxygen (Serrano-Silva et al. 2014; Knief 2015). Such C may then be taken up by primary producers (in the forms of carbonate and bicarbonate species) through the roots as the base of the food web (Limer et al. 2017; SKB 2019).

It is well-documented that in the case of ^14^C release into the biosphere, the majority of C in plants is fixed through photosynthesis from atmospheric ^14^CO_2_, and thus only a minor proportion of the C pool from organic matter is assimilated through roots (≤ 2%) (Amiro et al. 1991; Sheppard et al. 1991; Amiro and Ewing 1992; Milton et al. 1998; Avila and Pröhl 2008; van Dorp and Brennwald 2009; Hoch 2014; Mobbs et al. 2014; Limer et al. 2017). Despite proportionately lower uptake of C from organic matter than that of the atmosphere, many studies emphasized the importance of C uptake from soil to roots, especially in the case of release from waste disposal facilities (Majlesi et al. 2019; Ota and Tanaka 2019; Jyllilä 2022). In such scenarios, root uptake becomes more significant, especially when there is a relatively higher fraction of ^14^C in soil because of operational or accidental discharges in the soil system than that of the air. Furthermore, it has been suggested that root colonization of plants by mycorrhizae possibly facilitates the uptake of C from soil to plants (Taylor et al. 2004; Majlesi et al. 2019).

In aquatic ecosystems, available ^14^CO_2_ is exchanged between air and water by fixation of atmospheric C by phytoplankton, algae, and aquatic plants as dissolved inorganic carbon (DIC), primarily in forms of CO_2_ and bicarbonate species (Rasilo 2013; Limer et al. 2017; SKB 2019). Moreover, ^14^C in the soil (solution) can be transported to groundwater and aquatic environments in organic or inorganic forms. The ^14^C assimilated by plants can be deposited in bed sediment or returned to the water column through respiration. Aquatic plants, at the bottom of the food web, can be directly grazed by animals or indirectly contribute to the organic matter pool through root exudation or as plants’ leftovers (Chappuis et al. 2017). On the other hand, organic matter of allochthonous origin may also carry ^14^C in particulate (e.g., through sedimentation) and dissolved phase (e.g., from soil solution) to the aquatic environment. The ^14^C incorporated into organic matter may then be directly taken up by living matter. Moreover, methanogens use the available C pool to form CH_4_ under anoxic conditions. After the oxidation of CH_4_ to CO_2_ by methanotrophs at the oxic-anoxic interface, C may be taken up by the roots of vegetation, which are consumed by various species at higher trophic levels (Sanseverino et al. 2012).

The extent to which C can be transferred from sediment organic carbon (SOC) into biota is unclear. Therefore, because of limited data, uncertainties are still associated with the transfer of ^14^C from organic substrates into aquatic organisms. Although water is traditionally regarded as the primary nutrient pathway for many aquatic organisms, C distributed in the biosphere may ultimately enter the detritus pool and become part of the detritus-based ‘’brown food web’’(Evans-White and Halvorson 2017), where decomposers break down organic compounds, which are then used by flora and fauna. This is particularly important regarding the availability of inorganic C to primary producers in the form of DIC.

One of the main challenges in examining the contribution of SOC in organisms is how to distinguish it from atmospheric C. Artificial labeling of sediment with radioactive C isotopes is associated with risks to the environment or the laboratory staff. In this work, a novel approach based on ^14^C natural abundance was used to distinguish the sources of C between peat (in the following referred to as “sediment organic carbon or SOC”) and atmospheric C in aquatic plants. In this approach, the contribution of SOC was investigated, using peat samples from a cut-away peatland, where there was a very large difference in ^14^C/total C ratio between the modern atmosphere (100 pMC) and up to 8000-year-old leftover peat after peat extraction (38 pMC). The peat was highly depleted in ^14^C due to the low atmospheric concentration of ^14^C before nuclear weapon tests started in the 1950 s and the decay of ^14^C with time (Biasi et al. 2011). The present-day peat values are expected to be ≥ 100 pMC, while its δ^13^C signature is consistent with reference values, being − 26.8‰, compared to the atmospheric level (−8‰) (Convey et al. 2011). This approach allows calculating the proportion of SOC vs. the atmospheric C in aquatic plants.

The main aim of the study was to investigate and compare the transfer of SOC (^13^C and ^14^C) into roots and leaves of various aquatic plants in a microcosm study. However, the contribution of water as an important exposure pathway was also examined in the selected species. The selected plants included three common categories of aquatic vegetation: submergent (*Littorella uniflora*), emergent (*Lysimachia nummularia* and *Stachys palustris*), and free-floating (*Lemna minor* or common duckweed). These species are common and widely distributed in northern ecosystems, including Finland’s freshwaters. They are known as the source of food and protection for many species and are important for the integrity of aquatic ecosystems. However, the importance of these species is not only limited to aquatic environment; *L. minor* is used as feed for livestock and poultry, *L. nummularia* is a valuable plant in medicine for treatment of diseases and the nectar of *S. palustris* is attractive for bumblebees, which can further disperse the seeds of these plants because of pollination (Taylor and Rowland 2011; Forni and Tommasi 2016; Doğan 2022). The ^13^C isotopic signature was also used as a proxy for the uptake of ^14^C into plants to understand the contribution of SOC. Application of stable isotopes is generally preferred over the use of radiolabeling techniques, which are associated with potential hazards. The findings of the present work are important in producing more data on the uptake of ^14^C in aquatic flora for developing current radioecological models associated with ^14^C biosphere assessment.

## Materials and methods

### Study site and field sampling

The peat samples were collected in June 2023 from a peatland area in Linnansuo, the rural area of the city of Joensuu in Eastern Finland (N62° 53′ and E30° 43′). The peat was primarily used for supplying energy and later cultivated with two plant species, reed canary grass or RCG (*Phalaris arundinacea*) and Scots pine (*Pinus sylvestris*). The mean annual temperature is around 2 °C and the precipitation is 669 mm. The peatland is an ombrotrophic *Sphagnum fuscum* bog. The peat extraction included drainage by ditching, which began in 1978 and ended in 2001, when the thickness of the remaining peat decreased, ranging from 20 to 85 cm before the start of the cultivation. After the extraction of the upper layers of the peat, the lowermost layers, being naturally up to 8000 years old, and thus highly depleted in ^14^C, had become accessible (Biasi et al. 2011). The fraction of organic matter was high (approximately 71%). The average C content and pH were 39% and 4.5, respectively (for more information on the site see Biasi et al. 2008; Shurpali et al. 2008). The peat samples were collected to a depth of 50 cm by digging. They were transported to the university and stored at 4 °C. After that, the peat samples were homogenized and sieved at 4 mm to remove extra materials before the experiment.

### Plant samples

The plant samples (*Littorella uniflora*, *Lysimachia nummularia*, and *Lemna minor*) were purchased from a commercial supplier in Kuopio, Finland (Pieni Eläinkauppa). *Stachys palustris* was collected from Lake Kallavesi near the University of Eastern Finland, Kuopio campus (N62° 89′ and E27° 63′). The emergent and submerged species are perennial plants, but the duckweeds were purchased as plantlets. After the transportation of the plants to the university laboratory, they were immediately washed to remove sediment and other debris from roots and leaves. After that, all plants were added to the test system.

### Experimental set-up

The peat with a natural abundance of ^14^C was used as the substrate to examine the transfer of SOC into the plants. To establish the open chamber microcosm, 15-L aquariums (31 cm x 21 cm x 24 cm) were added with 500 g thin layer of peat (wet weight) at the bottom with the depth of approximately ≤ 1 cm and 4 L of artificial freshwater in the laboratory under 16:8 h light: dark photoperiod (2000 lumens LED lights; color temperature of 6500 K; under PPFD of 150–200 µmol m^− 2^ s^− 1^). The amount of peat was kept to the minimum level to avoid a decrease in pH and its possible detrimental effects on the plants. The water content of the peat was 91%. The temperature in the laboratory was 20 ± 1 °C and the microcosm was connected to the natural ventilation system. The artificial freshwater was made with milli-Q water according to OECD guidelines 203 (OECD 1992), which specifies standard concentrations of major ions (Ca^2+^, Mg^2^⁺, Na⁺, K⁺, Cl⁻) to achieve consistent freshwater chemistry and ionic composition corresponding to a total hardness of approximately 2.5 mmol L^− 1^ (Ca^2+^ + Mg^2+^) in the microcosm. The pH of the artificial freshwater was 7. The plants were then added to 0.01 M phosphate-buffered artificial freshwater (sodium phosphate dibasic and sodium phosphate monobasic, pH = 7). The addition of a buffer was necessary to avoid the decrease in pH level caused by the acidic nature of the peat. However, keeping pH at the desired level was challenging. Thus, the water in the test systems was changed every ten days to maintain the pH level circumneutral. The dissolved oxygen level, pH, and temperature were monitored during the entire experiment using the multi-parameter portable meter (multi-3620 IDS, Xylem Analytic, Germany). The total duration of the experiment was one month, and the treatments were established separately for each plant species (Table 1). The emergent species were extended above the water, the submerged species were fully underwater, and the floating plants partially covered the water surface. After the experiment, the plant samples were rinsed in milli-Q water, and the roots and the leaves were carefully separated.

**Table 1:** Experimental setup of microcosm study with naturally depleted peat in ^14^C and artificial freshwater. Values indicate the number of aquariums (number of individuals per aquarium) for each species

Plant species	Number of aquariums (number of individuals per aquarium)
*L. minor* (free-floating)	6 (N.D.) *
*L. uniflora* (submergent)	18 (2)
*L. nummularia* (emergent)	7 (4)
*S. palustris* (emergent)	6 (3)

N.D. not determined. *The number of individuals for *L. minor* was not available since they were purchased as solution bags (100 mL per bag). The individuals from five bags were added to one aquarium

### Analysis of samples

All the plant samples were oven-dried at 40 °C for 48 h to remove the moisture. Moreover, to obtain sufficient biomass for analysis of duckweeds, the whole plants without separation of roots and leaves were pooled together from one aquarium. The sediment samples were randomly collected from different tanks (*n* = 2 from the sediment of each emergent species, *n* = 3 from *L. minor* sediment and *n* = 3 from *L. uniflora* sediment; total = 10) and oven-dried similarly to the plant samples before analysis. 10 ml of water samples (*n* = 3 from each species sediment; total = 12) were also randomly collected and filtered by Whatman filter paper (pore size, 0.45 μm) and freeze-dried (Heto LyoLab 3000 Freeze Dryer, Thermo Fisher Scientific) for 48 h before and after the experiment. After sample treatments, they were prepared for the ^13^C analysis.

To determine the ^13^C signature and C content (%) of plants, 2–3 mg of the dried samples of roots (*n* = 10 individuals) and their corresponding leaves (*n* = 10 individuals) from each species were randomly selected and weighed into tin cups before and after the experiment. The analysis of duckweed was carried out for the whole plant (*n* = 10). Similarly, 2–3 mg of the sediment (*n* = 10) and water samples (*n* = 12) were placed into the tin cups for analysis. The samples were analyzed by an elemental analyzer isotope ratio mass spectrometry (EA-IRMS) (Thermo Finnigan, FLASH EA 1112, Milan, Italy), at the University of Eastern Finland, Kuopio campus.

For ^14^C analysis, randomly selected sediment samples (*n* = 3), and samples of submergent and emergent plants (the dried roots with their corresponding leaves; *n* = 3 for roots of each species and *n* = 3 for leaves of each species), as well as the whole duckweeds (*n* = 4) were transported to the Finnish Museum of Natural History, LUOMUS, Helsinki, Finland. All radiocarbon analyses of plant samples were carried out by accelerator mass spectrometer (AMS) with high precision (< 0.2%). The radiocarbon dating laboratory reported percent modern carbon (pMC) for all samples and provided the corresponding radiocarbon ages. The pMC is defined as a ratio of ^14^C/^13^C in the sample compared to the same ratio in 1950 standard (1950; Oxalic Acid II standard, NIST SRM 4990 C). All ^14^C measurements are normalized to the standard δ^13^C value of −25‰ to correct for isotopic fractionation (Stuiver and Polach 1977). This normalization is required because natural isotopic fractionation alters the ^14^C/^13^C ratio during biological and chemical processes. Correcting all samples to a common reference value (−25‰) removes these fractionation effects, allowing pMC values to reflect only radioactive decay and carbon-source mixing rather than isotopic discrimination.

1 $${\mathrm{Correcte}}{{\mathrm{d}}^{{\mathrm{14}}}}{\mathrm{C}}={\mathrm{Measure}}{{\mathrm{d}}^{{\mathrm{14}}}}{\text{C }} - {\text{ }}({\mathrm{8}}.0{\text{33 }} \times {\text{ }}({\delta ^{{\mathrm{13}}}}{{\mathrm{C}}_{\mathrm{S}}} - {\text{ }}\left( { - \,{\mathrm{25}}} \right)))$$

Where, 8.033 is a fixed number that relates δ^13^C to fractionation correction and δ^13^C_S_ is δ^13^C of the sample.

### Mycorrhizal colonization level

The total mycorrhizal colonization level in plants’ roots was determined, using the staining technique according to Giovannetti and Mosse (1980) (*n* = 4 for each species). After washing the plant roots, they were kept in 10% KOH for 24 h at room temperature, followed by rinsing them in 1% HCl before staining. The root samples were then stained with lactophenol blue stain (Diagnostica Merck, E. Merck, 64271 Darmstadt Germany) for 24 h at room temperature and rinsed overnight in deionized water to remove excess stain. The 1-cm long root pieces were mounted on a microscopic slide in groups of 10 and investigated by Motic AE21 inverted research microscope (Motic Microscopes, China) for the presence or absence of infection. The total mycorrhizal colonization level was calculated as the total number of infected roots divided by the total number of all roots in the sample × 100 and was expressed as a percentage.

### Data analysis

We applied a simple two-pool isotope mixing model because only two isotopically distinct sources of carbon were present in the microcosm (sediment vs. atmosphere/water). Bayesian models such as SIAR or MixSIAR are typically used when multiple sources and prior probability distributions are incorporated. In our system, the large isotopic contrast between sediment and atmospheric/water carbon and the low number of sources allow a deterministic two-source model to provide most appropriate and transparent approach, providing estimates with low parameter uncertainty.

Two-pool isotope mixing model was used as follows to estimate the relative contribution of the two sources (sediment vs. the atmosphere) in the plants:

2 $$\begin{gathered} {\mathrm{f1}}=({\text{pMC or corrected }}{\delta ^{{\mathrm{13}}}}{{\mathrm{C}}_{{\mathrm{SAMPLE}}}}-{\text{ pMC or corrected }}{\delta ^{{\mathrm{13}}}}{{\mathrm{C}}_{{\mathrm{SOURCE2}}}}) \hfill \\ \quad \quad /{\text{ }}({\text{pMC or corrected }}{\delta ^{{\mathrm{13}}}}{{\mathrm{C}}_{{\mathrm{SOURCE1}}}}-{\text{ pMC or corrected }}{\delta ^{{\mathrm{13}}}}{{\mathrm{C}}_{{\mathrm{SOURCE2}}}}) \hfill \\ \end{gathered}$$

3 $${\mathrm{f1}}\,+\,{\mathrm{f2}}\,=\,{\mathrm{1}}$$

where f1 = fraction of source 1, contributing to the sample, and f2 = 1-f1, which is the fraction of source 2, contributing to the sample. In this case, source 1 was the sediment, and source 2 was the atmosphere (as an alternative source of C). The δ^13^C and ^14^C values of the sediment samples were separately pooled together (using the arithmetic mean) after analysis, representing homogenous distribution of the organic matter in the whole aquatic environment. The δ^13^C and ^14^C values of the atmosphere used in the model were − 8‰ and 100 pMC, respectively. The current ^14^C activity in the atmosphere has returned to its natural level of 226 Bq kg^− 1^ C (100 pMC).

To assess whether peat mineralization altered the isotopic composition of carbon available in the water column, we also measured the δ^13^C of the dissolved carbon pool in water samples collected at the beginning and end of the experiment. In addition to the approach explained above, to estimate the fraction of C in *L. minor* and *L. uniflora*, initial δ^13^C signature of water (before the experiment) is also used as the second endmember. The use of water in the second approach is because the roots of *L. minor* have no access to bed sediment and are floating in the water, which can have a primary role in the uptake of C. However, it should be noted that the transfer of sediment-derived C may still take place through the decomposition of organic matter and the re-suspension of C in sediment particles into the water column. Furthermore, *L. uniflora*, as a fully submerged species, cannot directly assimilate C from the air, but rather indirectly absorbs atmospheric C dissolved in water through the gas exchange (i.e., from the DIC pool). This suggests that the δ^13^C signature of dissolved C can change because of the isotopic fractionation in water. Henceforth, to estimate the contribution of sediment-derived C in *L. uniflora*, only water was used as the second source of C. For this purpose, only ^13^C datasets were employed in the mixing model as no water samples were analyzed for ^14^C data. Moreover, the ^13^C signatures of the plant samples were corrected to a reference value of 20‰ for the fractionation caused by Rubisco enzyme (Farquhar et al. 1989). This correction was applied because Rubisco discriminates strongly against ^13^C during carbon fixation, causing plant organic matter to be approximately 20‰ more negative than the δ^13^C of the carbon source it assimilates (Farquhar et al. 1989). Adjusting the measured plant δ^13^C values by this fractionation allows the values to be used to estimate the isotopic composition of the assimilated carbon before enzymatic discrimination. This approach enables direct comparison of plant-derived δ^13^C with sediment and DIC endmembers in the mixing model.

To determine the rate of C uptake in the selected species, the contribution of SOC was normalized to plants’ C content, using the difference between the initial (C_i_) and final C content (%), (C_f_). This approach determines the C uptake in plant tissues during the experimental period:

4 $$\begin{aligned}&{\text{Plant C uptake from sediment}}\\&={\text{contribution of SOC }}\left( \% \right){\text{ }} \times {\text{ }}\left( {{{\mathrm{C}}_{\mathrm{f}}} - {\text{ }}{{\mathrm{C}}_{\mathrm{i}}}} \right),{\text{ }}\left( \% \right)\end{aligned}$$

For statistical analysis and to calculate the standard errors and 95% confidence intervals of f1 and f2, Isoerror version 4.1 was used (Phillips and Gregg 2001). The average values of plants (from each species), air, water and sediment concentrations with their corresponding standard deviations as well as the number of analyzed samples were included to estimate the standard errors and 95% confidence intervals. The contribution of SOC was considered statistically significant from zero contribution (e.g., no transfer from SOC) when 95% confidence intervals of f1 did not include zero. A paired sample t-test was used to compare the transfer of SOC between roots and leaves within individuals of each species. The difference was considered statistically significant when *P* ≤ 0.05. SPSS 29 for Windows (SPSS Inc., an IBM Company) was used for the independent sample t-test.

## Results

### Water characteristics

**Fig. 1:**
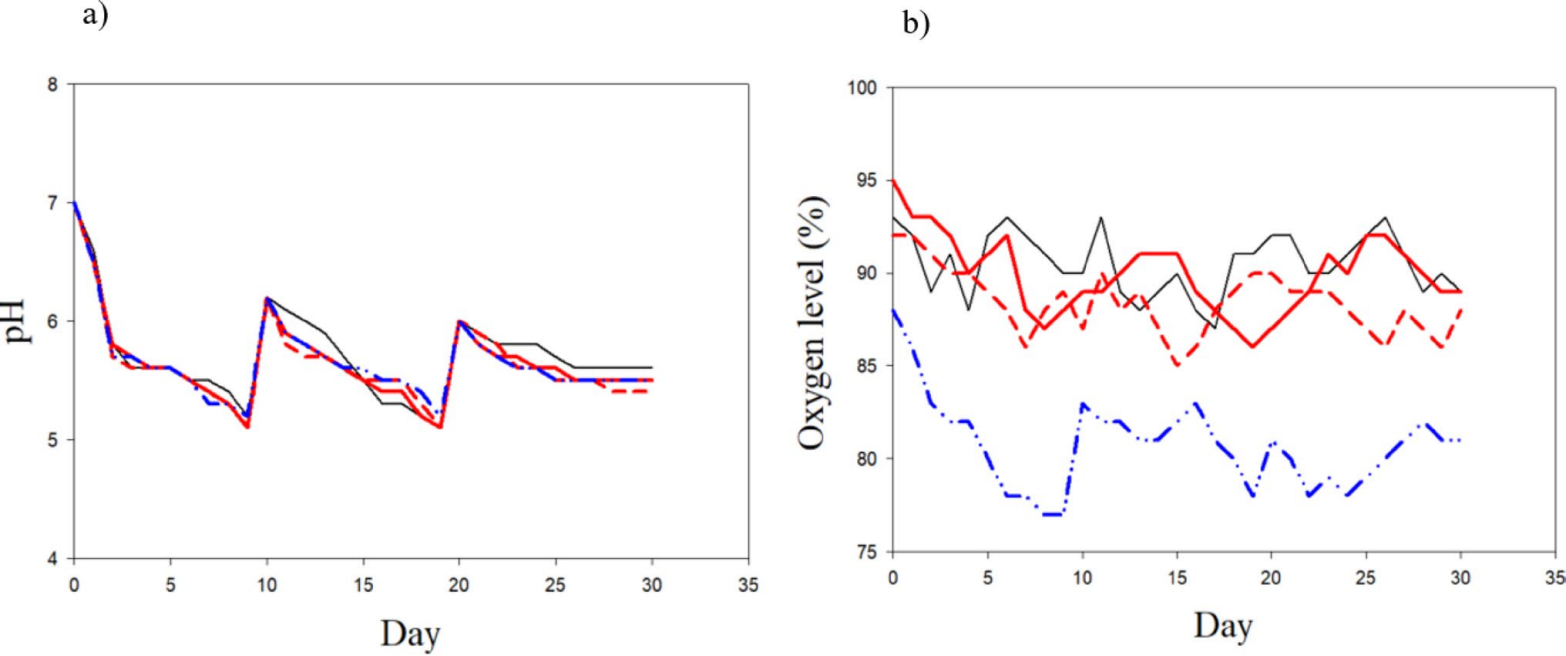
Characteristics of artificial freshwater (pH and oxygen levels) during the experiment in the treatments with the selected plants. The water was changed on days 10 and 20. *L. minor*= black line; *S. palustris* = red solid line; *L. nummularia* = red dashed line and *L. uniflora* = blue line

The pH value of water at the beginning of the experiment and after adding the buffer was 7. Nevertheless, the pH decreased to 6 after 48 h. After a continuous decrease in pH, it remained constant, ranging from 5 to 5.5 (Fig. 1). A similar trend was observed after each change of water, showing a pH level of 6 immediately after a few hours. The oxygen level in the overlying water was 77 to 95% and the water conductivity ranged from 1.1 to 1.6 mS/cm. The water temperature was 20 ± 1 °C during the entire experiment.

### Plants’ characteristics, isotopic analysis and mycorrhizal colonization of the roots

**Fig. 2:**
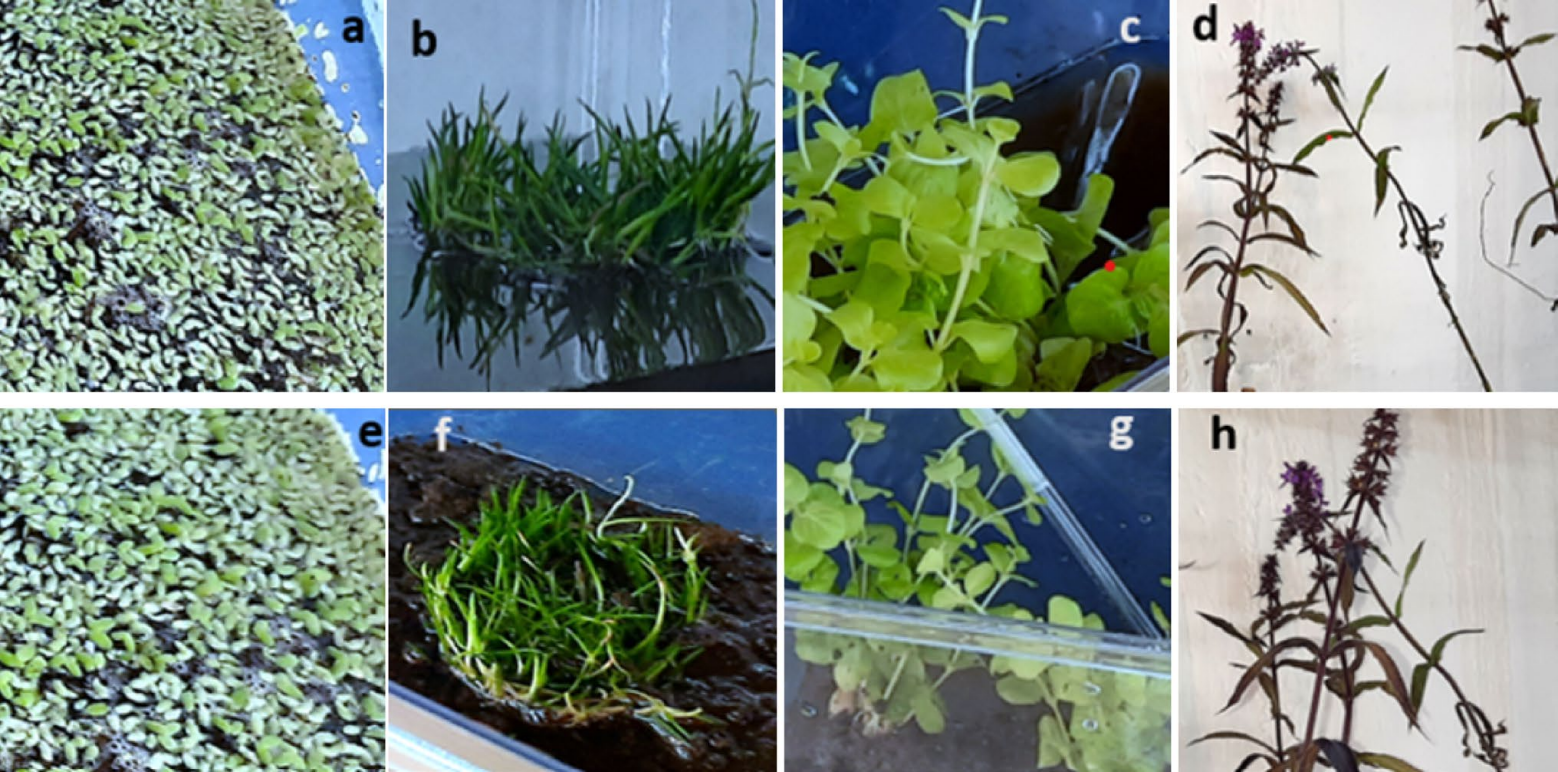
Physical appearance of the aquatic plants used in the experimental microcosm before (a-d; upper panel) and after (e-h; lower panel) the experiment. a and e = *L. minor*, b and f = *L. uniflora*, c and g = *L. nummularia* and d and h = *S. palustris*

The studied plants showed no visible signs of physical changes (e.g., change in biomass or appearance of new shoots, etc.) during the experiment (Fig. 2). The average δ^13^C signatures (± standard error of the mean, SEM) and ^14^C content of the sediment samples (pMC) were − 26.8‰ (± 0.08) and 38.4 (± 0.28), respectively (Table 2). The sediment samples were highly depleted in ^14^C, being equivalent to an age of 7688 years before the present (BP) as reported by the radiocarbon dating laboratory. The average δ^13^C signature (± SEM) of water samples before the experiment was − 9‰ (± 0.14), showing the similarity of their isotopic composition to the atmosphere (−8‰). Moreover, the water samples showed a similar δ^13^C value of −26.7‰ (± 0.11) to the sediment samples after the experiment. The average total C contents in water before and after the experiment were 1.02% (± 0.02) and 12% (± 1.24), respectively, being equivalent to 2.59 and 33 mg C L^− 1^.

Among the plant species, the average δ^13^C signatures ± SEM were mostly similar, ranging from − 31.9‰ ± 0.11 in *L. uniflora* leaves to −29.5‰ in the roots and the leaves of *S. palustris* (Table 2). The average ^14^C content ± SEM of the plants also showed similar results among the species, ranging from 91.5 pMC ± 1.31 in the leaves of *L. uniflora* to 99.2 pMC ± 0.41 in the roots of *S. palustris*. However, *L. minor* was the only species that was highly depleted in ^13^C and ^14^C, compared to the other plants, showing values of −34.9‰ ± 0.04 and 64.9 pMC ± 4.99, respectively. After calculating the corrected concentration of plant samples, δ^13^C signatures showed the average values of −14.9‰ for *L. minor*, −9.5‰ for *S. palustris* roots and leaves, −9.8‰ and − 9.6‰ for *L. nummularia* roots and leaves respectively, and − 11.7‰ for roots and − 11.9‰ for leaves of *L. uniflora*. Moreover, the lowest total C content (%C) in the plant samples was observed in *L. minor* from 30% ± 0.44 before the experiment to 32.8% ± 0.97 after the experiment.The highest values were detected in the leaves of *S. palustris* from 31.8% ± 0.52 before the experiment to 42.3% ± 1.01 after the experiment. In general, the emergent species (*S. palustris* and *L. nummularia*) showed slightly higher C content in the leaves than the roots, while for *L. uniflora* an opposite trend was observed.

**Table 2:** (Corrected) δ^13^C signature (‰), ^14^C content (expressed as percent modern carbon; pMC), and total C content (%C) in sediment and plant samples (before and after the experiment). SEM represents the standard error of the mean

Sample	δ^13^C ± SEM	Corrected δ^13^C ± SEM	pMC ± SEM	%C ± SEM^1^		%C ± SEM^2^
Sediment	−26.8 ± 0.08		38.4 ± 0.28	39.0 ± 2.57		39.0 ± 2.57
Water (initial)	−9.00 ± 0.14		N.D.	1.02 ± 0.02		N.A.
Water (end of experiment)	−26.7 ± 0.11		N.D.	N.A.		12.0 ± 1.24
*L. minor*	− 34.9 ± 0.04	−14.9 ± 0.04	64.9 ± 4.99	30.0 ± 0.44		32.8 ± 0.97
*S. palustris* roots	−29.5 ± 0.12	−9.5 ± 0.12	99.2 ± 0.41	31.3 ± 0.22		38.9 ± 0.51
*S. palustris* leaves	−29.5 ± 0.14	−9.5 ± 0.14	99.1 ± 0.33	31.8 ± 0.52		42.3 ± 1.01
*L. nummularia* roots	−29.8 ± 0.34	−9.8 ± 0.34	98.4 ± 0.10	30.3 ± 0.21		33.5 ± 0.21
*L. nummularia* leaves	−29.6 ± 0.14	−9.6 ± 0.14	99.1 ± 0.23	31.1 ± 0.42		36.3 ± 0.54
*L. uniflora* roots	−31.7 ± 0.04	−11.7 ± 0.04	91.9 ± 1.25	31.6 ± 0.33		39.9 ± 0.54
*L. uniflora* leaves	−31.9 ± 0.11	−11.9 ± 0.11	91.5 ± 1.31	31.4 ± 0.31		38.2 ± 1.85

N.D. not determined N.A. not applicable ^1^%C before the experiment and ^2^%C after the experiment

The results indicated mycorrhizal colonization only in the roots of *L. uniflora*. However, the average root colonization was rather small, being only 7.25% (Table 3). No root colonization was observed in other species.

**Table 3:** Average mycorrhizal colonization level (% of infected roots) and standard error of the mean (SEM) in *L. uniflora* roots

Root sample	L. uniflora	SEM
Sample 1	7.00	0.02
Sample 2	8.00	0.02
Sample 3	6.00	0.02
Sample 4	8.00	0.03
Mean	7.25	0.48

### Transfer of SOC into hydrophytes

**Fig. 3:**
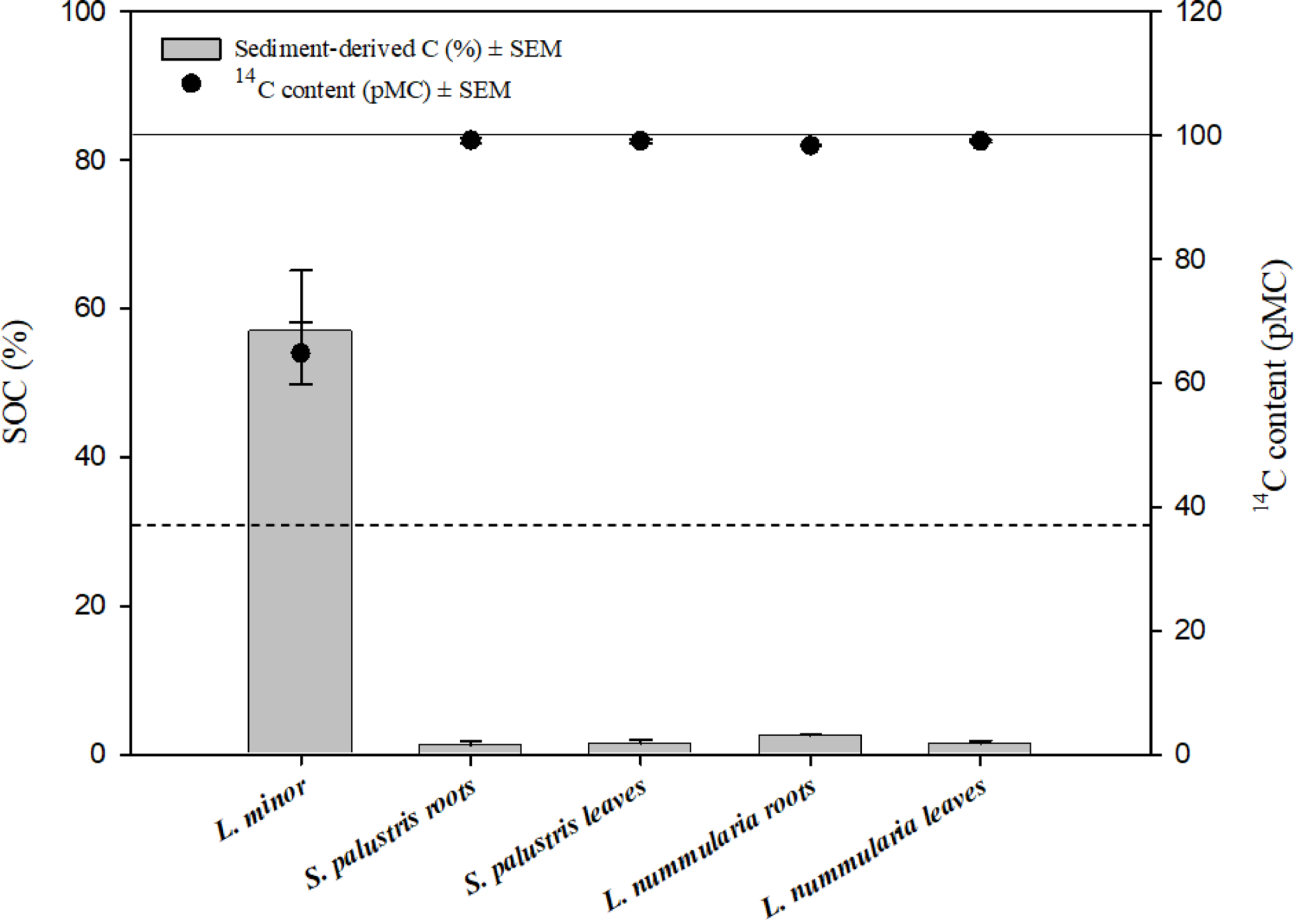
Contribution of sediment organic carbon (SOC in %) in aquatic plants, including ^14^C primary data (in pMC, percent modern carbon). The solid line represents atmospheric pMC (100) and the dashed line represents the pMC content of the sediment (38.4 ± 0.28). SEM=standard error of the mean. The transfer of SOC showed a statistically significant difference from zero contribution in all plants. The statistical analyses showed no significant difference in the transfer of SOC between the roots and the leaves of each species

Given the ^14^C content of the hydrophytes in Table 2, the mixing model results revealed the highest contribution of SOC in free-floating plants (*L. minor*), 57.1% ± 8.11 (Fig. 3). The transfer of SOC was significantly higher in *L. minor* than zero contribution. Moreover, the results showed that the contribution of SOC in the emergent species was 1.25% ± 0.65 and 1.41% ± 0.54 in the roots and the leaves of *S. palustris*, respectively, while it was 2.59% ± 0.16 in the roots and 1.46 ± 0.37 in the leaves of *L. nummularia*. The contribution of SOC in these species was significantly different from zero. The statistical analyses revealed no significant difference in the transfer of SOC between the roots and the leaves of each species (*P* = 0.85 for *S. palustris*, 0.21 and for *L. nummularia*).

**Fig. 4:**
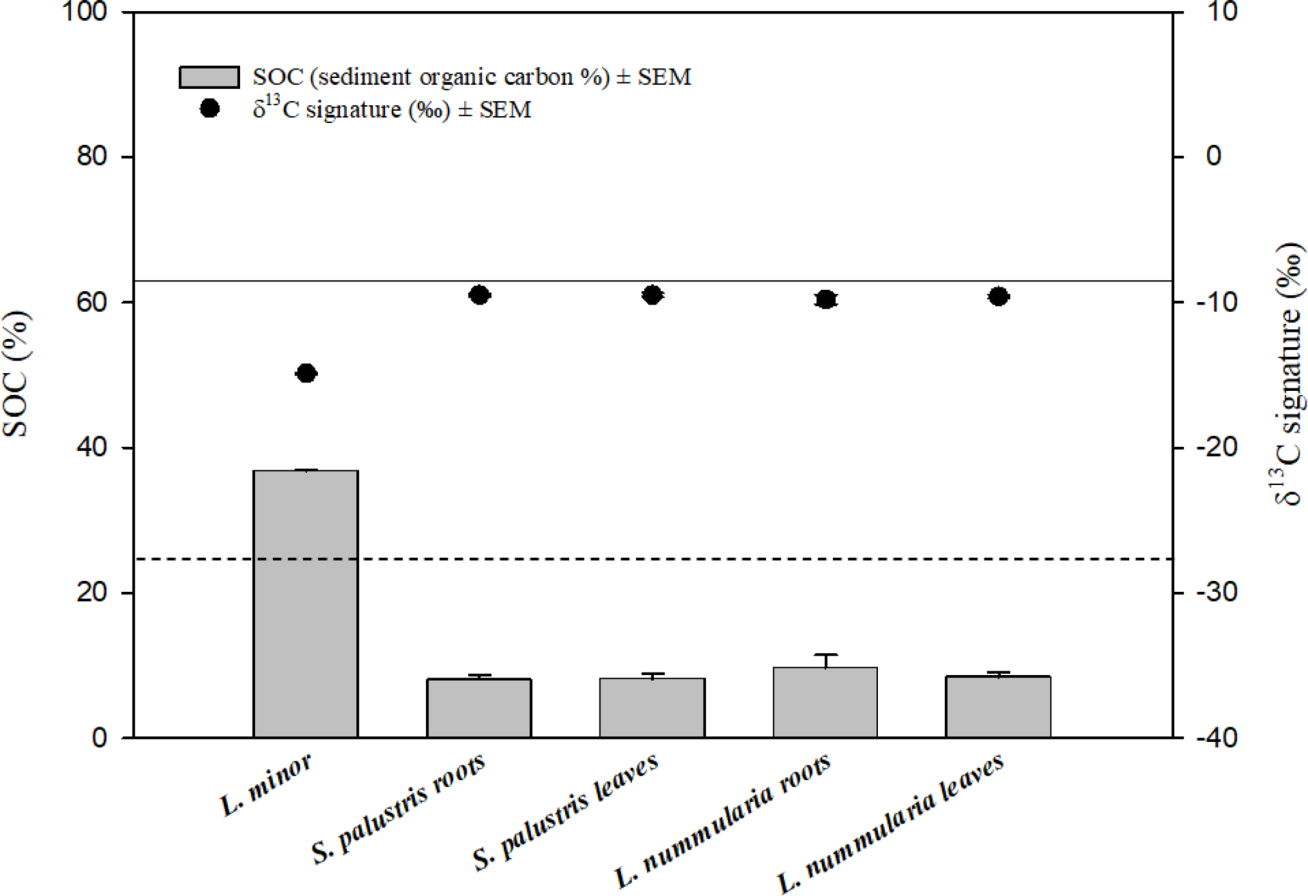
Contribution of SOC in aquatic plants, including δ^13^C signatures (‰). The solid line represents the atmospheric δ^13^C signature (−8‰) and the dashed line represents the δ^13^C signature of the sediment (−26.8 ± 0.08). SEM=standard error of the mean. The transfer of SOC showed a statistically significant difference from zero contribution in all plants. The statistical analyses showed no significant difference in the transfer of SOC between the roots and the leaves of each species

**Fig. 5:**
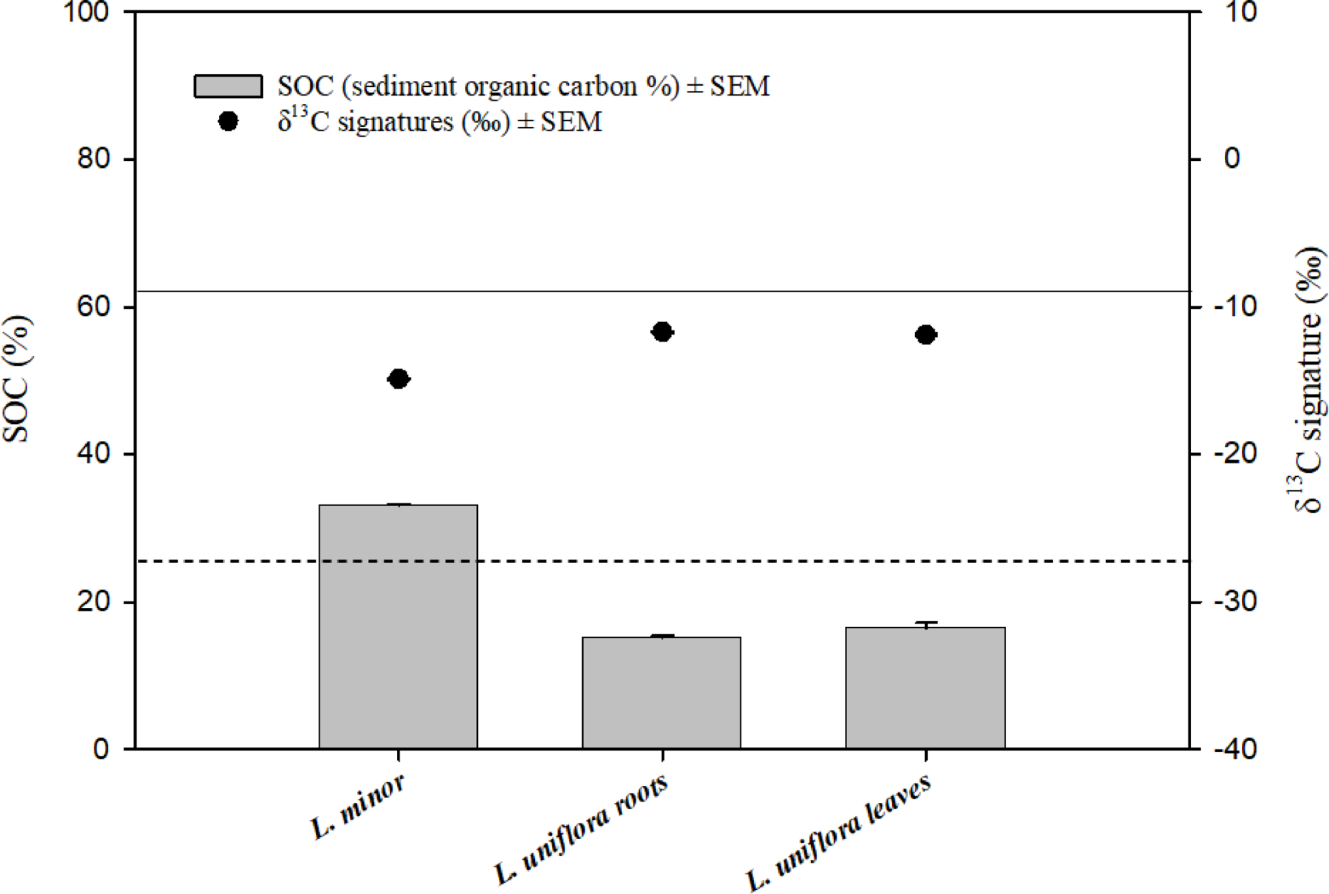
Contribution of SOC in aquatic plants with corresponding δ^13^C signatures (‰), using water as the second end member of the mixing model. The solid line represents the water δ^13^C before the experiment (−9‰) and the dashed line represents the δ^13^C signature of the sediment (−26.8 ± 0.08). SEM=standard error of the mean. The transfer of SOC showed a statistically significant difference from zero contribution in all plants. The statistical analyses showed no significant difference in the transfer of SOC between the roots and the leaves of *L. uniflora*

Mixing model results using ^13^C datasets showed a similar trend to the ^14^C data in the transfer of SOC into the hydrophytes. The transfer was the highest in *L. minor*, followed by the emergent plants (*S. palustris and L. nummularia*) (Fig. 4). The transfer of C, originating from the sediment, was 36.7% ± 0.21 in *L. minor*, whereas the contribution of SOC was found to be 8.02% ± 0.61 and 8.12% ± 0.75 in the roots and in the leaves of *S. palustris*, respectively. Similar results were also observed from the contribution of SOC to the roots (9.66% ± 1.78) and the leaves (8.35% ± 0.73) of *L. nummularia.* No significant difference was found in the transfer of SOC between the roots and the leaves within the species (*P* = 0.91 for *S. palustris* and 0.48 for *L. nummularia*). Moreover, using the alternative approach (water as the second source) showed that the transfer of SOC was 15.2% ± 0.24 and 16.5% ± 0.63 in the roots and in the leaves of *L. uniflora*, respectively (Fig. 5). No significant difference was observed in the transfer of SOC between the roots and the leaves of these species (*P* = 0.11). The same approach used for *L. minor* only slightly changed the outcome of the mixing model, revealing 33.1% ± 0.22 transfer of SOC in the floating plants (Fig. 5).

### Uptake of SOC normalized to total C content (%) into the hydrophytes

Applying Eq. 4 to determine the uptake of C into the hydrophytes, showed lower uptake of SOC in plant tissues (Table 4). The results showed less than 1% of C uptake in emergent species and slightly more than 1% in submerged plants. The floating plant (*L. minor*) showed the highest C uptake among the hydrophytes (1.61%). In general, the C uptake was slightly higher, using ^13^C data in the plants studied. However, *L. minor* was the only species that showed slightly greater C uptake, using ^14^C data.

**Table 4:** Uptake of SOC in the studied hydrophytes, including the difference between initial and final C content (%), (C_f_-C_i_). The lower and upper range in *L. minor* indicate the uptake of sediment-derived C using water and air as the alternative source in the mixing model, respectively

Plant	(C_f_-C_i_) (%)		Plant uptake (%), ^13^C data	Plant uptake (%), ^14^C data
*L. minor*	2.8		0.93–1.03	1.61
*S. palustris* roots	7.6		0.61	0.09
*S. palustris* leaves	10.5		0.85	0.14
*L. nummularia roots*	3.2		0.32	0.08
*L. nummularia leaves*	5.2		0.43	0.07
*L. uniflora* roots	8.3		1.26	N.A.
*L. uniflora* leaves	6.8		1.12	N.A.

N.A. not applicable

## Discussion

In this work, the isotopic mixing model was used to investigate the transfer of SOC into aquatic plants. Using ^14^C natural abundance and stable isotope of C (^13^C) as a proxy for uptake of ^14^C demonstrated to be reliable for partitioning C sources between the highly depleted sediment and other sources (atmosphere and water). Because the system contained only two isotopically distinct endmembers, the two-source mixing model provided clear partitioning of sediment versus atmosphere/water-derived carbon, allowing robust interpretation of the observed transfer patterns. The difference in the ^14^C signatures between the sources (depleted sediment and more enriched air and water) is ideal for tracking the proportion of C from the underground in various environmental samples. This is particularly important in assessing the consequences of ^14^C release from underground facilities and nuclear industries into organic matter and its transportation to the aquatic environment.

Although both ^13^C- and ^14^C-based mixing models revealed the same general pattern of SOC transfer among the studied hydrophytes, slight quantitative differences between the two isotopes were expected. This is because δ^13^C values retain the effects of biological isotopic fractionation from diffusion, carbonate equilibrium processes, and enzymatic discrimination by Rubisco (Farquhar et al. 1989), whereas radiocarbon activities (expressed as pMC) were mathematically normalized for isotopic fractionation during AMS analysis. According to the standard convention (Stuiver and Polach 1977), all ^14^C measurements were corrected to a reference δ^13^C value of −25‰ to remove isotopic fractionation and allow direct comparison of samples. As a result, pMC values reflect only radioactive decay and mixing between sources, while δ^13^C values, even after applying the Rubisco correction, retained additional biological and environmental fractionation-related variability. These methodological differences explain why the ^13^C- and ^14^C-based mixing models produced slightly different estimates, even though the overall trends remained consistent.

The results of this study showed the contribution of SOC in the selected species. The transfer was approximately 60% in *L. minor*, about 15–17% in *L. uniflora*, and 1–3% in the emergent species (*S. palustris* and *L. nummularia*). Although the rate of uptake was slightly different using ^13^C datasets (33–37% in *L. minor* and 8–10% in the emergent species), ^13^C and ^14^C results indicated a similar trend in the uptake, being the highest in the floating species and the lowest in the emergent plants. The findings revealed that only *L. minor* preferred a relatively higher contribution of SOC than the atmosphere. Among the selected hydrophytes, *L. minor* was also the only species which roots were floating in the water, as the roots of the other plants were deposited in the bed sediment. This suggests that after the decomposition of SOC, the uptake rate of DIC (as CO_2_ or bicarbonate species) is more efficient in suspended roots of *L. minor* and its photosynthetically active fronds, consistent with previous findings that DIC availability and sediment type strongly influence hydrophytes carbon acquisition and physiological traits (Li et al. 2023). There is also data from a previous study, showing a similar contribution of SOC (approximately 60%) originating from the same peat in DOC (Majlesi et al. 2020). This means that if the organic C in water is decomposed to inorganic C, it becomes readily available to floating plants such as *L. minor* as the transfer of SOC to the water column was demonstrated in Table 2 (from 1% at the beginning of the experiment to 12% at the end of the experiment) (Leifeld and Menichetti 2018). This was also evidenced by similar ^13^C values between sediment and water samples after the experiment, suggesting biological activities and transfer of C to the water environment. The high transfer of SOC in *L. minor* might also be related to the pH level, which mostly ranged from 5 to 5.5 during the experiment. At this range, DIC dominantly prevails in the form of CO_2_, which is preferable to plants over bicarbonate species, which are mostly available at pH ≥ 7 (Vuorinen et al. 1989; Maberly and Gontero 2017). The study reported that bicarbonate assimilation is usually slow and energetically less favorable to plants as it needs to be catalyzed to CO_2_ before fixation. *L. uniflora* was the other species, in which a higher contribution of SOC was observed than the emergent plants. *L. uniflora* is a benthic species, which is fully submerged in water. These plants have developed mechanisms to adapt and survive under extremely low concentrations of inorganic C by uptake and retention of C from the sediment through permeable root surfaces (Søndergaard and Sand-Jensen 1979; Keeley 1998; Grolander 2016; Li et al. 2023; Thorne et al. 2023). Moreover, the lacunar system in these hydrophytes facilitates the gas exchange and transfer of large amounts of CO_2_ from the sediment (Hostrup and Wiegleb 1991; Colmer 2003; Yamauchi et al. 2018). Although this study showed slight root colonization of *L. uniflora* by mycorrhizae (7%), they can possibly contribute to the transfer of C from the organic matter. Previous studies also reported the role of mycorrhizae in the possible transfer of C from organic matter to terrestrial and aquatic plants, including *L. uniflora* (Taylor et al. 2004; Shah 2014; Fusconi and Mucciarelli 2018; Majlesi et al. 2019). These attributes of *L. uniflora* may explain the higher transfer of SOC than the emergent species, whose leaves are well above the water surface and can efficiently take up atmospheric C through stomata on the epidermis. Recent work by Pronin et al. (2024, 2025) has shown that *L. uniflora* tends to display higher (less negative) δ^13^C values under acidic conditions, suggesting a strong isotopic response to pH, DIC availability, and the use of specialized carbon acquisition strategies in CO_2_-limited softwater environments. In our experiment, pH decreased from neutral (approximately 7) to moderately acidic (5–5.5.5), which is consistent with conditions where such mechanisms may become increasingly relevant. However, the δ^13^C values of *L. uniflora* in our system (approximately − 32‰) were more depleted than those reported by Pronin et al. (2025) likely due to the strong influence of ^13^C-depleted sediment-derived CO_2_ and limited renewal of water-column DIC in the small microcosms. Therefore, while our primary focus was on the partitioning between sediment-derived and atmospheric/water carbon, the observed SOC contribution in *L. uniflora* likely is supported by pH-dependent shifts in DIC speciation and carbon-uptake pathways described by Pronin et al. (2024, 2025) rather than being the only driver of isotopic variation.

The contribution of SOC within species was similar between the roots and the leaves. This was approximately 15–17% in submerged *L. uniflora* and about 1–3% in *S. palustris* and *L. nummularia*. The similarity of uptake in these plants may suggest upward transportation of C from roots to shoots through xylem tissues (Enoch and Olesen 1993; Hoch 2014; Sinha et al. 2022). In such processes, the internal CO_2_, taken up by roots can move rapidly through the xylem to leaves via transpiration stream (Livingston and Beall 1934; Amiro and Ewing 1992; Bloemen et al. 2013). Of the C assimilated via the transpiration stream, some proportion is lost, while other C content is used as sugar for leaves and plants’ biomass (Hoch 2014). Although such a mechanism is absent in fully submerged plants due to lack of stomata, upward transportation of water and nutrients may still take place through the process of guttation, in which water is removed via hydathodes (Pedersen 1993; Singh 2016). Another possible reason for the similar uptake of SOC between the roots and the leaves can be related to the presence of aerenchyma in the selected species. In general, partially and fully submerged hydrophytes possess well-developed tissue structures (aerenchyma), which contain air spaces between and within roots and shoots (Takahashi et al. 2014). Such structure can facilitate the rapid exchange of gases, including CO_2_, allowing hydrophytes to survive under stressful conditions to promote photosynthesis (Takahashi et al. 2014; Yamauchi et al. 2018; Ikonen 2022).

In this study, we also applied the mixing model by using water δ^13^C signature as an alternative end member of C in floating and benthic species. This is particularly relevant for *L. uniflora*, which assimilates C from water rather than the atmosphere. Using this approach, the contribution of SOC was found to be 15–17%. However, no isotopic fractionation is expected to occur, assuming that equilibrium is reached between the air and the water and thus pMC content remains the same in the water as in the air. Such simplifying assumptions are commonly used in radioecological models for predicting the transfer of radionuclides during controlled and constant discharges of radionuclides from nuclear facilities to the biosphere (IAEA 2010). In this study, ^14^C content of the water samples was not analyzed, but similar ^14^C value of *L. uniflora* (92 pMC, Table 2) to the atmospheric value may suggest comparable ^14^C content (and small isotopic fractionation) between the water and the atmosphere. Therefore, such assumptions might be useful to estimate the approximate proportion of C from the water. Under this assumption, if we had used the pMC value of the air (100 pMC) as a proxy for the water in the mixing model, we would have obtained similar transfer of SOC in the roots and the leaves of *L. uniflora* (13–14%). The results of this work also showed that because of the similarity of δ^13^C signatures between water (−9‰) and atmosphere (−8‰), using water as the second endmember only marginally changed the transfer of SOC in *L. minor* (33%). Similar δ^13^C signatures between air and water suggested only minor isotopic fractionation of C with dissolution of atmospheric CO_2_ in the water. It should be noted for further experiments that these results can vary depending on such environmental parameters, that is, the amount of C in the water and its incorporation with atmospheric CO_2_.

Given the contribution of SOC normalized to C content in the hydrophytes, it was possible to estimate the rate of C uptake in the species. Despite the significant transfer of SOC to the plants, only < 2% of C was found in the plant’s tissues. This is consistent with broad literature, reporting the central value of ≤ 2% of C from organic matter (soil/sediment) as a minor pathway in plants (Grolander 2016 and references therein; Majlesi et al. 2025 and references therein). These findings suggest that the large proportion of the carbon in the form of DIC, which originates from the sediment, is either lost through plants’ respiration or may go under degassing process before uptake by the plants and thus only a minor proportion becomes truly available to the hydrophytes. Furthermore, the results of this study showed less than threefold C content in the dissolved phase than SOC, suggesting a lower proportion availability of DIC for plant uptake (Table 2).

No visible signs of physical changes were observed in the studied species. This might be because of the short duration of the experiment since the studied hydrophytes were mostly perennial plants (except for duckweeds), which may last several months to years to add biomass. The slow growth of duckweeds and other plants can also be attributed to sub-optimal conditions in the laboratory environment.

Only *L. minor* showed some variation in ^14^C content among the plants studied. To obtain sufficient biomass for the analysis, all the individuals of *L. minor* from one aquarium were pooled together. As individuals have different needs for C in biomass production, this may cause variation in the uptake of SOC in these species. Furthermore, pH was an important factor in the uptake of C by *L. minor*, which mostly ranged from 5 to 5.5. Keeping the pH between 6 and 7 was challenging because of the acidic pH of the sediment. Overall, the pH level in Finland’s freshwaters is mostly circumneutral (Toivonen and Huttunen 1995mäläinen and Huttunen 1998). However, shifts in pH levels (i.e., down to ≤ 4) may occur in boreal waters during various seasons because of changes in temperature, ice-covered period, and water chemistry (Edén et al. 1999; Järvinen et al. 2002; Saarinen et al. 2010; Sutela et al. 2010). Another limitation of the study was the duration of the experiment, which was one month. Although the duration is consistent with the average longevity of *L. minor*, other species are known as perennial plants, which may live for two or more years. Considering the perennial plants’ lifespans, keeping them under laboratory conditions for a long period was impossible as they cannot tolerate allochthonous sediments with relatively acidic pH (Maranho and Gomes 2024; Xiong et al. 2024). Hence, the longer duration of the experiment under optimal conditions might change the uptake rate. In general, there are limitations in carrying out laboratory studies since many other variables such as water and sediment physico-chemical properties, the need of plants for different light intensities and seasonal variations might affect the uptake process. However, such studies are important, especially when finding ecosystems with large differences in natural abundance or distinct ^14^C activity is challenging. Moreover, data on the transfer of ^14^C into hydrophytes is limited and the findings of this study can significantly contribute to radioecological modeling of ^14^C. As the next step, carrying out field studies and in-situ measurements are recommended for aquatic plants.

## Conclusion

In this work, the transfer of SOC into three categories of aquatic plants was investigated in a microcosm experiment. This is important for current ^14^C radioecological modeling as data on aquatic ecosystems are limited. The findings of this study are crucial concerning the possible release of ^14^C from nuclear facilities into aquatic ecosystems. The results, using ^13^C and ^14^C datasets showed the transfer of C from the organic substrate as one of the sources into free-floating plants (33–60%), submerged (15–17%), and emergent species (1–9%). We also found that due to similar δ^13^C signatures between the water and atmosphere, applying the water as an alternative end member only slightly changed the transfer of SOC in floating species (33%). The contribution was 37%, using the atmosphere as the second source. Despite the contribution of SOC, a smaller amount of C (< 2%) was taken up by the plants studied. Moreover, no significant difference was observed in the transfer of SOC in the roots and in the leaves within the species. The findings particularly highlighted the importance of floating plants in the (indirect) uptake of sediment-derived C from the water or the air. Further research on the transfer of ^14^C into hydrophytes particularly under field conditions is recommended.

## Data Availability

All the original data in this work are included in the article.
